# Evaluation of new classifications of N descriptor in non-small cell lung cancer (NSCLC) based on the number and the ratio of metastatic lymph nodes

**DOI:** 10.1186/s13019-016-0456-5

**Published:** 2016-04-14

**Authors:** Konrad Pawełczyk, Marek Marciniak, Piotr Błasiak

**Affiliations:** Department of General Thoracic Surgery, Wroclaw Thoracic Surgery Centre, Wroclaw Medical University, Wroclaw, Poland

**Keywords:** Metastatic lymph nodes, TNM classification, Non-small cell lung cancer

## Abstract

**Background:**

The aim of the study was to evaluate the prognostic power of new classifications of N descriptor created basing on the number (NLN) and the ratio of metastatic lymph nodes (RLN) in NSCLC compared to the current classification (CLN).

**Methods:**

The data of 529 patients with NSCLC operated with the intention of radical resection, were analyzed. The new categories of N descriptor were created as follows: 1) NLN - median number of metastatic nodes was 3, thus in NLN0 the number of metastatic nodes =0, in NLN1 1-2, in NLN2 ≥ 3, 2) RLN - median ratio (number of metastatic lymph nodes to all nodes removed) was 12.4 %, thus in RLN0 the ratio was 0, in RLN1 < 13 %, in RLN2 > 13 %. The prognostic value of each classification was calculated on the basis of hazard ratios defined in multivariate Cox proportional hazard model.

**Results:**

The new classifications of N descriptor turned out to be an independent strong prognostic factor (*p* <0.001) with a 5-year survival rate NLN0-62 %, NLN1-39 %, NLN2-26 % and RLN0-62 %, RLN1-37 % and RLN2-26 %. For 5-year survival rates in CLN0-62 %, CLN1-42 %, CLN2-24 % (*p* < 0.001), a higher prognostic value of new classifications was not demonstrated, the hazard ratio amounted to 2.22, 2.08, 2.49 for NLN2, RLN2 and CLN2 respectively.

**Conclusion:**

Despite the significantly high prognostic power, the new classifications cannot be considered superior over CLN. There are some deficiencies in the current classification, therefore further studies on its improvement are needed.

## Background

Despite the high heterogeneity of N descriptor, its classification in NSCLC has remained unchanged since 1987, when the fourth edition of the TNM classification was published [1]. The heterogeneity of N descriptor refers mainly to metastases in mediastinal lymph nodes (N2) where we can identify patients with a single metastatic node within one node station, several metastatic nodes in one node station, multi-level N2 disease and patients with “bulky disease”. According to many reports, the prognosis in these groups, as well as an approach to a surgical management being a part of multimodality therapy or disqualification from the operation, may be different [2–4]. The heterogeneity of N descriptor causes that the spread of neoplastic processes to mediastinal lymph nodes, frequently excluding intrapulmonary nodal groups and a pulmonary hilum (skipping metastasis) is unsymmetrical and difficult to predict. According to some reports, the prognosis of patients with N2 but negative N1 is better than of patients with a positive N2 and N1 simultaneously [5]. The classifications of N descriptor in NSCLC appearing successively, however, are based on the location of lymph nodes and assume the symmetrical spread of the cancer.

Therefore, due to the differences in the prognosis associated with heterogeneity of N2 descriptor, it should be considered whether to classify lymph nodes in NSCLC depending on the number of metastatic nodes or on the ratio of the number of resected nodes to metastatic nodes. A similar assumption has been adopted for instance in staging a colorectal, gastric and breast cancer in which the number of metastatic lymph nodes has been considered one of the elements staging N descriptor [6]. It is also suggested that the ratio determining the number of metastatic lymph nodes, particularly relating to a breast, bladder, colorectal cancer may have a significant prognostic value in the classification of N descriptor [7–9].

The aim of the paper is to evaluate the prognostic value of classifications based on the number of metastatic lymph nodes (NLN) and on the ratio of the number of resected nodes to metastatic nodes (RLN) compared to the current classification of N descriptor (CLN) in NSCLC.

## Methods

The data of 700 patients with lung cancer subsequently operated with the intention of radical resection, were analyzed. All patients were treated surgically between 1 January 2007 and 1 July 2009 in one, high volume thoracic surgery center. Data for statistical analyzes were obtained from the National Lung Cancer Registry. To eliminate factors which may additionally affect or do affect the prognosis, 92 patients after limited resections (wedge resection or segmentectomy), 7 patients with carcinoid tumor, 7 patients with small cell lung cancer, 14 patients with R1 resection, 3 patients with M1 and 13 perioperative deaths were excluded from the study. In addition, 35 patients with removed nodes <6 were excluded because the removal of at least 6 nodes is recommended to perform proper staging of N descriptor [10]. Finally, a group of 529 patients remained whose data were further analyzed.

Before taking a decision on a surgical treatment, all the patients had a chest radiography, chest computed tomography (CT), bronchoscopy, abdominal ultrasound and CT/MRI of the central nervous system, in case of neurological symptoms. Positron emission tomography (PET-CT) was performed in case of doubts, before the invasive diagnosis of mediastinal lymph nodes and in case of the diagnosis of mediastinal and peripheral changes in the suspicion of metastases. In case of suspicion of metastases to mediastinal lymph nodes during preoperative imaging (>1 cm diameter in a short axis) a videomediastinoscopy or a transtracheal/transbronchial biopsy was performed without ultrasound guidance (TBNA). Endobronchial ultrasound (EBUS) and endoscopic oesophageal ultrasound (EUS) were not performed as there were just introduced into the diagnosis of mediastinal lymphadenopathy. The surgical treatment in the studied group of patients included anatomical resection of the lung parenchyma (lobectomy, bilobectomy, pneumonectomy) and systematic mediastinal lymph node dissection with “en bloc” resection of right paratracheal 2R and 4R nodes. Nodes of 4 L, 5, 6, 7, 8, 9, 10 and 11 groups were removed separately. Intrapulmonary nodes of 12 group were removed together with a lobe, then carefully resected and evaluated by a histopathologist. The mediastinal lymph nodes were resected “en bloc” with the fatty tissue of mediastinum wherever it was possible to avoid defragmentation and were divided on the operating table, then each lymph node was sent for histopathological examination in a separate box. If the lymph node was disintegrated during harvesting the pieces were sent to evaluation in one box as a one lymph node. Pathological staging was evaluated on the basis of the current seventh edition of the TNM, all the stages were revised from the pathology reports and the Polish National Lung Cancer Registry.

The postoperative follow-up included radiological examination every 3 months for the first two years, then every 6 months for 3 next years, and once a year afterwards. Every 6 months, a control chest CT scan was performed. Median follow-up for the entire group was 61 months.

New categories of N descriptor were created in the following manner:

1. the classification based on the number of metastatic lymph nodes (NLN)
  Median number of metastatic lymph nodes was 3 (1 to 25). The median enables to create a boundary line between new NLN1 descriptor and NLN2. Thus, in patients with NLN0 descriptor the number of metastatic lymph nodes = 0, with NLN1 the number of metastatic lymph nodes <3 and with NLN2 ≥ 3 metastatic nodes.
2. the classification based on the ratio of metastatic lymph nodes (RLN)
  The ratio determines the quotient of metastatic lymph nodes to all nodes removed during the operation. The median of the calculated ratios was 12.4 % (2.2 % to 70 %). For patients with a RLN0 descriptor the ratio was 0, for patients with RLN1 < 13 %, for patients with RLN2 > 13 %.

The prognostic value of each classification was calculated on the basis of hazard ratios defined in the multivariate Cox proportional hazard model. The survival curves were plotted using Kaplan-Meier method and the differences in survival were determined using a log-rank test. Wilcoxon and χ^2^ tests were used to compare the clinical data. *P* <0.05 was considered statistically significant. STATISTICA software, version 10, StatSoft Inc. was used for statistical analyses.

## Results

Patient records including demographic, surgical and pathological data are presented in Table 1. On average, during the operation 23.4 nodes were resected (6 – 71). Among 529 patients included in the study, 377 (71.3 %) had no metastases in the resected lymph nodes. Postoperative chemotherapy was used in 45 patients with N1 descriptor (72 %) and in 56 patients with N2 (62 %), furthermore postoperative radiotherapy was used in 15 patients with N2 descriptor (17 %). Finally 36 patients (23 %) with N1 or N2 disease did not receive adjuvant therapy mainly because of inadequate performance status after surgery or other contraindications or a refusal of the further treatment.

**Table 1:** Patient characteristics and survival according to the current and new nodal classifications

Variables	Number of patients	5-year survival (%)	95 % CI	*p* value
Age				
≤68	279	59.5	56.2–63.0	<0.001
>68	250	46.4	43.6–49.4	
Sex				
Male	377	50.4	47.9–53.0	0.016
Female	152	60.5	56.0–65.5	
Histology				
Adenocarcinoma	251	50.2	47.2–53.4	0.1
Other	278	56.1	52.9–59.5	
Surgery				
Lobectomy	457	55.1	52.7–57.7	0.01
Pneumonectomy	72	41.7	37.1–46.8	
pT				
pT1a	72	72.2	65.0–80.2	<0.001
pT1b	69	78.3	70.9–86.4	
pT2a	219	54.8	51.3–58.5	
pT2b	69	46.4	41.1–52.3	
pT3	93	23.7	21.7–25.8	
pT4	7	28.6	18.8–43.4	
CLN				
CLN0	377	62	59.1–65.2	*p* < 0.001
CLN1	62	42	37.0–47.5	
CLN2	90	24	22.3–26.7	
NLN				
NLN0	377	62	59.1–65.2	*p* < 0.001
NLN1	70	39	34.3–43.3	
NLN2	82	26	23.3–28.2	
RLN				
RLN0	377	62	59.1–65.2	*p* < 0.001
RLN1	76	37	33.0–41.1	
RLN2	76	26	23.8–29.1	

*CI* confidence interval, *CLN* current nodal classification, *NLN* new classification based on the number of metastatic lymph nodes, *RLN* new classification based on the ratio of metastatic to resected lymph nodes

In 9 patients (1.7 %) a tumor was resected with the chest wall, in 11 patients (2.1 %) bronchial sleeve resection was performed, in 4 (0.7 %) artery sleeve resection, in 11 (2.1 %) wedge resection of another lobe on the same side. Mean greatest dimension of a tumor was 40.6 mm.

**Fig. 1:**
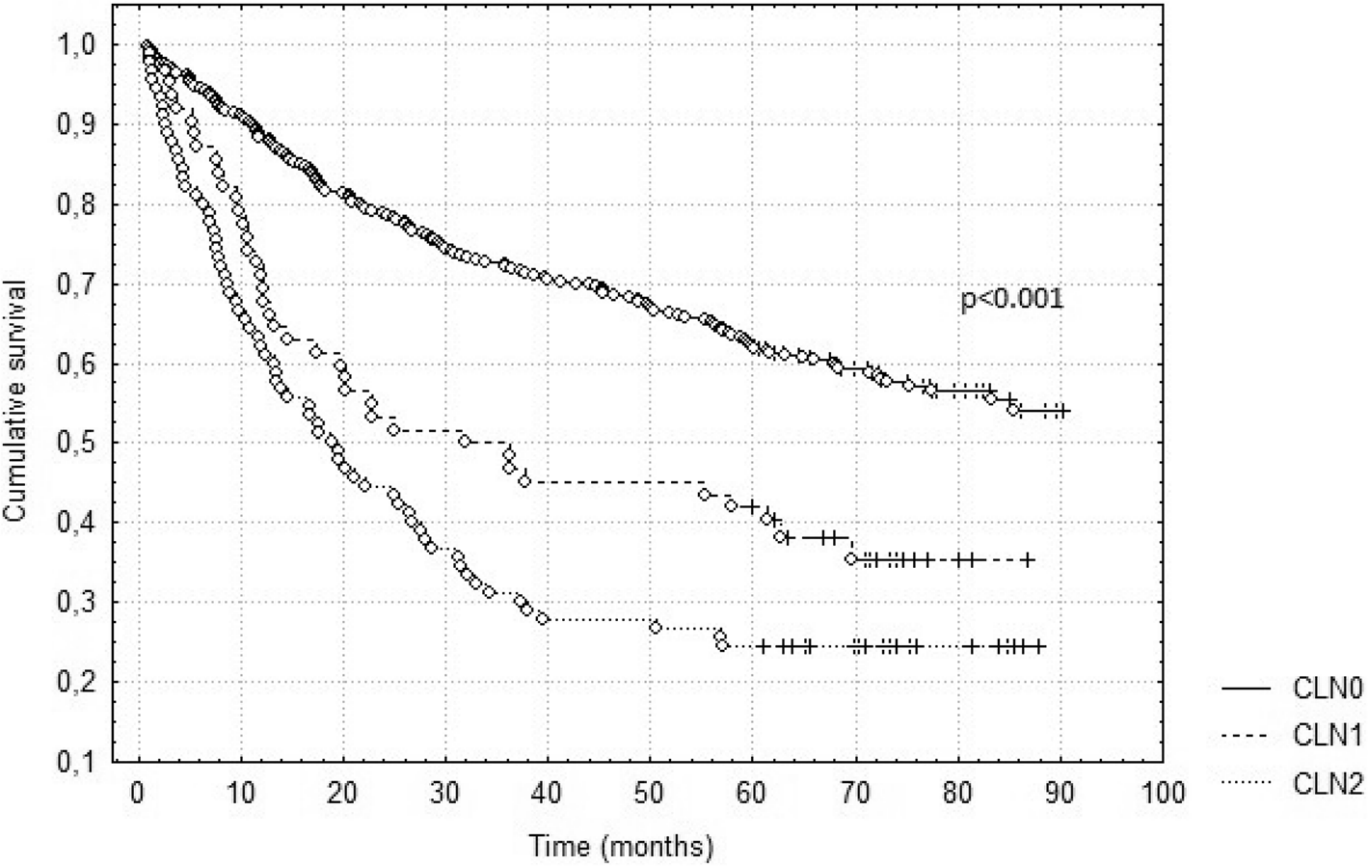
Survival according to current nodal classification (CLN)

**Fig. 2:**
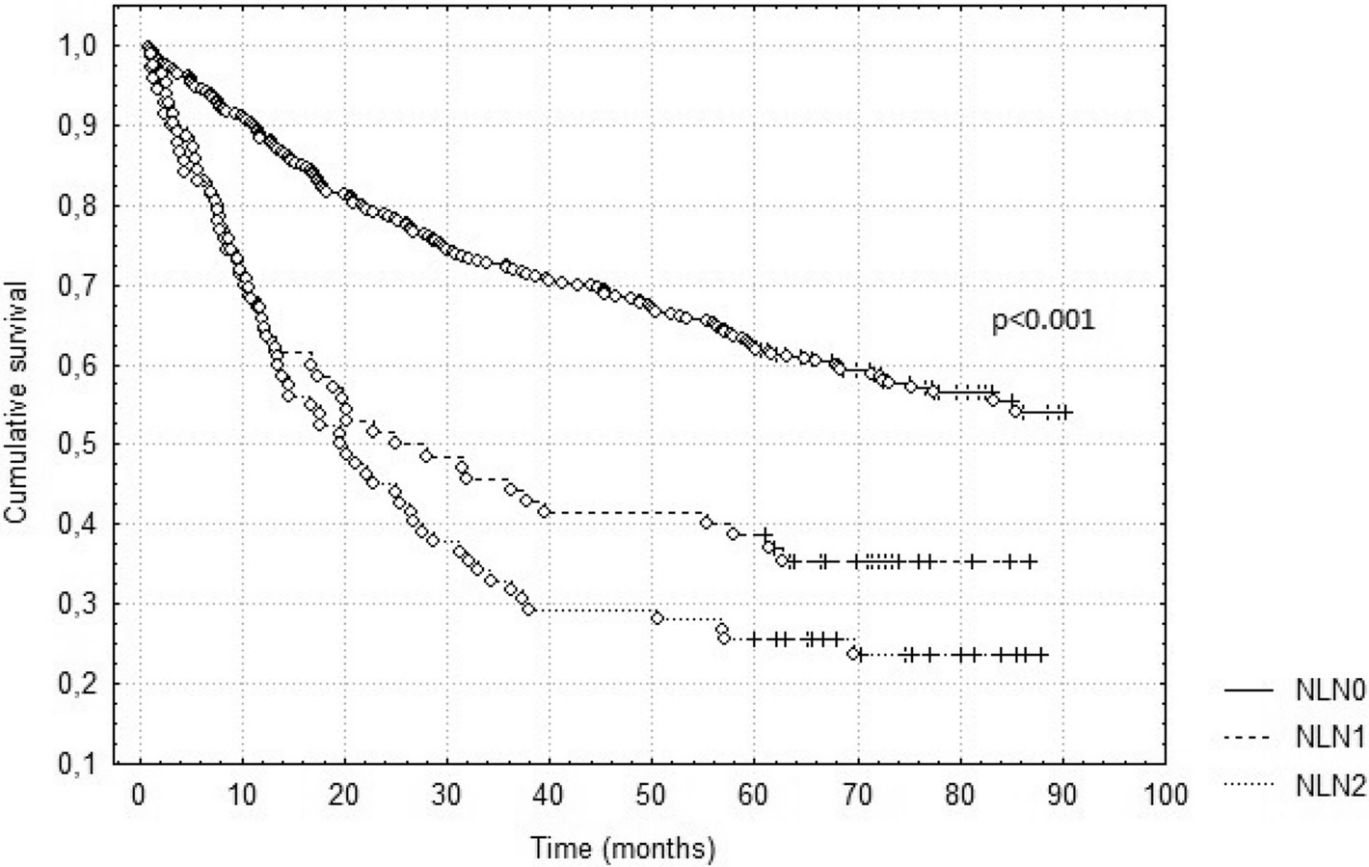
Survival according to new classification based on the number of metastatic lymph nodes (NLN)

**Fig. 3:**
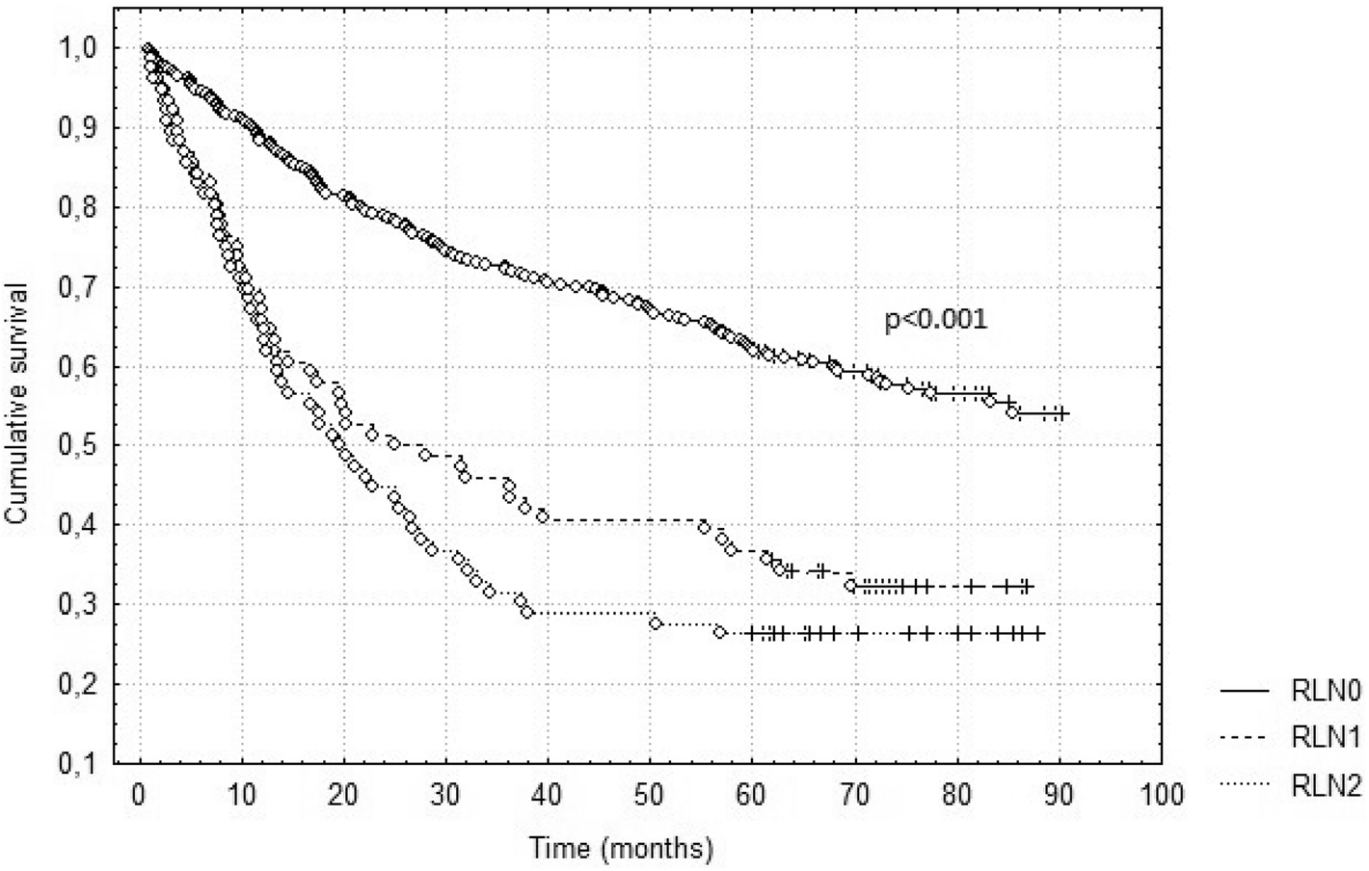
Survival according to new classification based on the ratio of metastatic lymph nodes (RLN)

The survival according to the current and suggested nodal classifications is presented in Table 1 and the survival curves according to CLN, NLN and RLN in Figs. 1, 2 and 3. All the studied classifications turned out to have a strong prognostic value (*p* <0.001). In order to evaluate the prognostic power of each classification, multivariate analysis was performed including all the factors analyzed univariately. Each of the studied nodal classifications was included in the multivariate analysis separately and values of hazard ratios in each analysis were compared. The data presented in Table 2 show that despite the small differences, the hazard ratio of the current N2 descriptor is the highest and amounts to 2.49 in comparison with NLN2 (HR = 2.22) and RLN2 (HR = 2.08). Each of the studied classifications in the multivariate analysis turned out to be a statistically significant prognostic factor but none of the newly studied classifications is superior over the current one. In the multivariate analysis it was also found that age (*p* < 0.001), tumor histology (*p* = 0.032) and pathological T descriptor (*p* < 0.001) are strong independent prognostic factors in the evaluation of overall survival of the patients.

**Table 2:** Comparison of hazard ratios of the current and new nodal classifications included in the multivariate analysis

Variables	HR	*P* value
CLN		
CLN1	1.49	0.025
CLN2	2.49	<0.001
NLN		
NLN1	1.74	0.001
NLN2	2.22	<0.001
RLN		
RLN1	1.90	<0.001
RLN2	2.08	<0.001

*CLN* current nodal classification, *NLN* new classification based on the number of metastatic lymph nodes, *RLN* new classification based on the ratio of metastatic to resected lymph nodes, *HR* hazard ratio

## Discussion

Metastases to mediastinal lymph nodes are currently indisputable and one of the strongest prognostic factors in a group of patients treated surgically because of non-small cell lung cancer [11]. Despite the high variability in N2 descriptor including patients with micrometastases or “bulky disease” and patients with a single metastatic node or multiple metastases in many nodal stations, the classification of N descriptor has remained unchanged for several editions of TNM. Despite the much better methods of the preoperative evaluation of mediastinal lymph nodes, in approximately 10–14 % of patients N2 disease is still found in a postoperative material [12, 13]. Moreover, many reports show that the prognosis of operated patients with N2 descriptor may considerably vary in the final histopathological evaluation due to the heterogeneity of metastases to mediastinal lymph nodes [4, 14]. The last modifications of the TNM system in certain malignancies such as colorectal, gastric and breast cancer involving only an anatomical classification of N descriptor incline to the similar approach to the classification of N descriptor in a lung cancer.

In this study, an effort was made to validate possible changes in the TNM system assuming the importance of the number of metastatic lymph nodes or the ratio of metastatic nodes to removed nodes in staging NSCLC. A homogeneous group of patients was selected, consecutively operated in a high-volume center, after the anatomical resections of lung parenchyma and the intraoperative removal at least 6 lymph nodes in order to verify the importance of the non-anatomical classification of N descriptor. The extent of lymph node resection raises a lot of discussions, however, there is no doubt that at least a systemic lymph node sampling is needed for staging N descriptor and most authors recommend mediastinal lymphadenectomy [10, 15, 16]. In literature there is no agreement regarding the minimum number of lymph nodes to be removed. It is recommended by ESTS to remove at least six nodes, according to the data compiled from the SEER database 11–16 nodes, according to Doddoli et al. 10 nodes and according to Wu et al. at least 15 nodes [10, 17–19]. In our study during the operation 23.4 lymph nodes were removed (10.6 % of patients had less than 11 nodes removed) which seems to be enough to stage N descriptor accurately.

In our study we do not confirm the hypothesis that assumes the better prognostic value of staging N2 descriptor based on the number of metastatic nodes or the ratio determining their proportions. While studying a group of patients after systematic lymph node dissection, it was found that the highest hazard ratio of the evaluation of N2 descriptor refers to the current classification of N2 descriptor. Despite the small differences between the various classifications, it cannot be stated that adding new elements to the classification of N descriptor in the new 8th edition of TNM will bring prognostic benefits. In the last years, a few studies have been published which assume that the effect of the number of removed lymph nodes or the ratio determining the number of metastatic nodes on survival is significant and better than the current classification [20–23]. However, these studies relate to long periods of observations mainly from the 80’s and 90’s of the last century, when the approach to preoperative staging, the extent of mediastinal lymphadenectomy, the evaluation of mediastinal nodes, the removal of metastatic lymph nodes from mediastinum (even “bulky disease”), the use of neoadjuvant chemotherapy for metastases to mediastinal lymph nodes were slightly different from that one in the middle of the last decade. Perhaps before we start to look for a better classification of N2 descriptor including its heterogeneity we should consider how to systematize and standardize the removal of lymph nodes in patients treated surgically because sometimes, even in one thoracic surgery center, the extent of lymphadenectomy may vary completely [24]. The studies, in which a higher prognostic value of metastatic nodes has been proved, assume the division of N descriptor into several subgroups depending on the ranges into which metastatic lymph nodes were divided [20, 21, 23]. Aiming at a simple method of classification of N descriptor, in our study we tried to determine a dividing line of metastatic nodes defined by the median. In our study, the median was 3 metastatic nodes, in a not much bigger population a similar dividing line was defined by Matsuguma (median 2 nodes) [22]. In studies on large populations, the median value is likely to change only a little and will range between 2–3 metastatic nodes. In order to avoid further development of the TNM system with numerous subgroups of N descriptor and making it more complex and less clear, it is justified taking into consideration the value of median in further study on the significance of the number of metastatic lymph nodes. The suggested division of the number of metastatic lymph nodes into groups: 1–3, 4–6, >6 [21], 1–4, 4–14, >14 [23], 1–3, 4–6, 7–9, ≥10 [20] can be found in literature. It seems that even basing on the studies on large populations, it will be difficult to establish several dividing lines of the number of metastatic nodes, as well as, it is currently difficult to define the minimum number of nodes that must be removed to perform appropriate staging of N descriptor. In addition, regardless of the size of nodes in imaging examinations, many of the centers have introduced minimally invasive techniques such as EBUS and EUS to routinely preoperative staging of N descriptor. This will probably cause that the chances to diagnose a several or even more metastatic lymph nodes in patients in the postoperative evaluation will be considerably reduced. However, it should be noted that although the prognostic power is weaker than the current one, the new classifications are also a significant and strong prognostic factor in a surgically treated NSCLC (*p* <0.001) in the evaluation of both N1 and N2 descriptors. Therefore it seems that we should not erase their significance. The study was currently carried out on a relatively small population which is its limitation. Though it is possible that studies on bigger populations will allow to include the ratio or the number of metastatic lymph nodes to the current classification system of N descriptor as its complement but not as a complete substitute.

It should be also taken into account that the current classification of N descriptor based on the location of nodes is used not only in the postoperative but also in the preoperative evaluation as it is easy to evaluate the location of nodes in imaging studies before the planned surgical treatment. When introducing new rules for the classification of N descriptor, there are some discrepancies between clinical and pathological staging of a disease. The complexity and difficulty of the pathological classification of lymph nodes resulting from the heterogeneity of N descriptor cause that in the future neither a location, quantity nor a ratio of metastatic nodes will be important in the decision on the adjuvant treatment. Probably the evaluation of nodes for novel molecular tumor cell markers determines a tumor aggressiveness, a status of N descriptor as well as, whether the patient should be treated after surgery or observed [25, 26]. In each case however, it will be necessary to remove an appropriate number of lymph nodes for pathological diagnostics.

## Conclusion

In conclusion, despite the significant prognostic power, it cannot be assumed that new classifications defining N descriptor are superior over the currently one. Nevertheless, it should be borne in mind that there are some deficiencies in the current classification, therefore further studies on its improvement are needed, regarding both the planned adjuvant treatment as well as, raising the rank of removing a proper number of lymph nodes to stage a disease appropriately.

## Abbreviations

CI: confidence interval
CLN: current nodal classification
HR: hazard ratio
NSCLC: non-small cell lung cancer
NLN: new classification based on the number of metastatic lymph nodes
RLN: new classification based on the ratio of metastatic to resected lymph nodes

## References

[1] Hermanek P, Sobin LH, International Union Against Cancer (UICC) (1987). TNM Classification of malignant tumours.

[2] Lorent N, De Leyn P, Lievens Y, Verbeken E, Nackaerts K, Dooms C (2004). Long-term survival of surgically staged IIIA-N2 non-small-cell lung cancertreated with surgical combined modality approach: analysis of a 7-year prospective experience. Ann Oncol.

[3] Detterbeck F (2008). What to do with “Surprise” N2?: intraoperative management of patients with non-small cell lung cancer. J Thorac Oncol.

[4] Decaluwe H, De Leyn P, Vansteenkiste J, Dooms C, Van Raemdonck D, Nafteux P (2009). Surgical multimodality treatment for baseline resectable stage IIIA-N2 non-small cell lung cancer. Degree of mediastinal lymph node involvement and impact on survival. Eur J Cardiothorac Surg.

[5] Riquet M, Assouad J, Bagan P, Foucault C, Le Pimpec BF (2005). Skip mediastinal lymph node metastasis and lung cancer: a particular N2 subgroup with a better prognosis. Ann Thorac Surg.

[6] Sobin LH, Gospodarowicz M, Wittekind C (2009). UICC TNM Classification of malignant tumours.

[7] Vinh-Hung V, Verschraegen C, Promish DI, Cserni G, Van de Steene J, Tai P (2004). Ratios of involved nodes in early breast cancer. Breast Cancer Res.

[8] Herr HW (2003). Superiority of ratio based lymph node staging for bladder cancer. J Urol.

[9] Peschaud F, Benoist S, Julié C, Beauchet A, Penna C, Rougier P (2008). The ratio of metastatic to examined lymph nodes is a powerful independent prognostic factor in rectal cancer. Ann Surg.

[10] De Leyn P, Lardinois D, Van Schil P, Rami-Porta R, Passlick B, Zielinski M (2007). European trends in preoperative and intraoperative nodal staging: ESTS guidelines. J Thorac Oncol.

[11] Osarogiagbon RU (2012). Predicting survival of patients with resectable non-small cell lung cancer: Beyond TNM. J Thorac Dis.

[12] Cho HJ, Kim SR, Kim HR, Han JO, Kim YH, Kim DK (2014). Modern outcome and risk analysis of surgically resected occult N2 non-small cell lung cancer. Ann Thorac Surg.

[13] Cerfolio RJ, Bryant AS, Ojha B, Eloubeidi M (2005). Improving the inaccuracies of clinical staging of patients with NSCLC: a prospective trial. Ann Thorac Surg.

[14] Van Meerbeeck JP, Surmont VF (2009). Stage IIIA-N2 NSCLC: a review of its treatment approaches and future developments. Lung Cancer.

[15] Darling GE, Allen MS, Decker PA, Ballman K, Malthaner RA, Inculet RI (2011). Number of lymph nodes harvested from a mediastinal lymphadenectomy: results of the randomized, prospective American College of Surgeons Oncology Group Z0030 trial. Chest.

[16] Vielva LR, Jaen MW, Alcácer JA, Cardona MC (2014). State of the art in surgery for early stage NSCLC-does the number of resected lymph nodes matter?. Transl Lung Cancer Res.

[17] Ludwig MS, Goodman M, Miller DL, Johnstone PA (2005). Postoperative survival and the number of lymph nodes sampled during resection of node-negative non-small cell lung cancer. Chest.

[18] Doddoli C, Aragon A, Barlesi F, Chetaille B, Robitail S, Giudicelli R (2005). Does the extent of lymph node dissection influence outcome in patients with stage I non-small-cell lung cancer?. Eur J Cardiothorac Surg.

[19] Wu YC, Lin CF, Hsu WH, Huang BS, Huang MH, Wang LS (2003). Long-term results of pathological stage I non-small cell lung cancer: validation of using the number of totally removed lymph nodes as a staging control. Eur J Cardiothorac Surg.

[20] Nwogu CE, Groman A, Fahey D, Yendamuri S, Dexter E, Demmy TL (2012). Number of lymph nodes and metastatic lymph node ratio are associated with survival in lung cancer. Ann Thorac Surg.

[21] Fukui T, Mori S, Yokoi K, Mitsudomi T (2006). Significance of the number of positive lymph nodes in resected non-small cell lung cancer. J Thorac Oncol.

[22] Matsuguma H, Oki I, Nakahara R, Ohata N, Igarashi S, Mori K (2012). Proposal of new nodal classifications for non-small-cell lung cancer based on the number and ratio of metastatic lymph nodes. Eur J Cardiothorac Surg.

[23] Lee JG, Lee CY, Park IK, Kim DJ, Park SY, Kim KD (2008). Number of metastatic lymph nodes in resected non-small cell lung cancer predicts patient survival. Ann Thorac Surg.

[24] Osarogiagbon RU, Allen JW, Farooq A, Berry A, O’Brien T (2011). Pathologic lymph node staging practice and stage-predicted survival after resection of lung cancer. Ann Thorac Surg.

[25] Nordgård O, Singh G, Solberg S, Jørgensen L, Halvorsen AR, Smaaland R (2013). Novel molecular tumor cell markers in regional lymph nodes and blood samples from patients undergoing surgery for non-small cell lung cancer. PLoS One.

[26] Ji HF, Pang D, Fu SB, Jin Y, Yao L, Qi JP (2013). Overexpression of focal adhesion kinase correlates with increased lymph node metastasis and poor prognosis in non-small-cell lung cancer. J Cancer Res Clin Oncol.

